# Single Hydrogel Particle Mechanics and Dynamics Studied by Combining Capillary Micromechanics with Osmotic Compression

**DOI:** 10.3390/gels9030194

**Published:** 2023-03-03

**Authors:** Kalpit J. Bakal, Andreas M. A. O. Pollet, Jaap M. J. den Toonder, Hans M. Wyss

**Affiliations:** 1 Microsystems Section, Department of Mechanical Engineering, Eindhoven University of Technology, 5600 MB Eindhoven, The Netherlands; 2 IDEAS Institute, Zhejiang University, Hangzhou 310058, China; 3 Institute for Complex Molecular Systems, Eindhoven University of Technology, 5600 MB Eindhoven, The Netherlands

**Keywords:** hydrogels, osmotic shock, creep test, gel mechanics, poroelasticity, gel swelling

## Abstract

Hydrogels can exhibit a remarkably complex response to external stimuli and show rich mechanical behavior. Previous studies of the mechanics of hydrogel particles have generally focused on their static, rather than dynamic, response, as traditional methods for measuring single particle response at the microscopic scale cannot readily measure time-dependent mechanics. Here, we study both the static and the time-dependent response of a single batch of polyacrylamide (PAAm) particles by combining direct contact forces, applied by using Capillary Micromechanics, a method where particles are deformed in a tapered capillary, and osmotic forces are applied by a high molecular weight dextran solution. We found higher values of the static compressive and shear elastic moduli for particles exposed to dextran, as compared to water ($K_{\mathrm{Dex}}\approx 63$ kPa vs. $K_{\mathrm{water}}\approx 36$ kPa, and $G_{\mathrm{Dex}}\approx 16$ kPa vs. $G_{\mathrm{water}}\approx 7$ kPa), which we accounted for, theoretically, as being the result of the increased internal polymer concentration. For the dynamic response, we observed surprising behavior, not readily explained by poroelastic theories. The particles exposed to dextran solutions deformed more slowly under applied external forces than did those suspended in water ($\tau_{\mathrm{Dex}}\approx 90$ s vs. $\tau_{\mathrm{water}}\approx 15$ s). The theoretical expectation was the opposite. However, we could account for this behaviour by considering the diffusion of dextran molecules in the surrounding solution, which we found to dominate the compression dynamics of our hydrogel particles suspended in dextran solutions.

## 1. Introduction

Hydrogels and hydrogel particles can exhibit a remarkably complex response to external stimuli, such as light [1,2], pH [3,4], temperature [5,6] and osmotic pressure [7]. For instance, the pH sensitive hydrogel, 2-hydroxyethyl methacrylate (HEMA)–acrylic acid (AA) [8], expands in volume for higher pH, and shrinks for lower pH, of the surrounding solution. Another interesting example is the temperature-sensitive hydrogel, poly(N-isopropylacrylamide) (pNIPAM), which undergoes a volume phase transition in response to changes in temperature [6] and shows a dip in its Poisson ratio and compressive modulus *K* near the lower critical solution temperature LCST [5]. Even chemically simple hydrogels, such as polyacrylamide (PAAm), can exhibit a surprisingly complex mechanical response to external stimuli. PAAm hydrogel particles can exhibit transient deswelling–reswelling behavior when exposed to an osmotic shock [7] or an overshoot in the volume upon swelling in a polymer solution [9].

Environmental variables, such as pH and temperature, are found in the human body, thus, pH and temperature sensitive hydrogels find applications in drug delivery systems. These drugs are encapsulated inside hydrogels and can be released by external stimuli, such as temperature [10,11,12,13,14,15] and pH [16,17,18,19].

Characterization of the mechanical properties of hydrogel particles at the single particle level is important for a range of different products and applications. For example, the single particle elastic properties and time scale of stress relaxation are of central importance in size exclusion chromatography, since the flow of fluid around the particles that make up the column compresses the particles and leads to modifications of the interstitial pore size [20,21]. Consequently, the fluid resistivity of the whole column increases nonlinearly with increase in the applied flow rate, depending on the time after which flow is applied [20]. Hydrogel particles are also frequently used as model systems to mimic the behavior of cells in biological systems, and to study processes, such as blood flow or transport of cells through biological tissue, where the single particle mechanical properties are of key importance.

However, it is difficult to characterize the mechanics of hydrogel particles at the single particle level, since traditional mechanical testing equipment does not operate at the low levels of force required for probing their response. Probing the mechanics of these materials, therefore, requires specialized techniques, such as atomic force microscopy (AFM) [22], osmotic compression [23,24,25], micropipette aspiration [26], or Capillary Micromechanics [27]. Another, more fundamental, issue is the interpretation of the measured mechanical response of hydrogel particles. True material properties should depend only on the properties of the bulk material, independent of the volume, or the shape, of the piece of material tested. However, for hydrogel particles, the characteristic time scales for compression or swelling depend critically on the size of the particles; so, indeed, it is not straightforward to characterize their viscoelastic response within the framework of material properties, such as frequency-dependent viscoelastic moduli $G^{\prime }\left(\omega \right)$ and $G^{\prime \prime }\left(\omega \right)$.

Previous work on single hydrogel particle mechanics has, therefore, mainly focused on measuring the static elastic properties of single particles in equilibrium, after the particle size has fully equilibrated to the externally applied stresses. Nevertheless, the dynamical response of hydrogel particles has been studied previously, as in the osmotic shock measurements by Sleeboom et al. [7], where, in order to apply a step-wise increase in osmotic pressure, the hydrogel particles were first captured in fluidic traps inside a microfluidic device with water as the background fluid. Subsequently, the background fluid was changed to a polymer solution to apply an osmotic shock to the particles and the resultant compression and swelling of the hydrogel particle was then recorded. Capillary Micromechanics, a method where particles are deformed in a tapered glass capillary under the influence of an applied pressure difference, could, in principle, also be used for time-dependent measurements, but previous work has focused on measuring static mechanical properties of hydrogels [5,27,28], core shell particles [29] and cells [30,31].

In this paper, we systematically study the response of a single batch of polyacrylamide (PAAm) hydrogel particles to external stresses that are applied by a combination of direct contact forces (using Capillary Micromechanics) and osmotic forces (through osmotic compression). We study the static response and time-dependent response of our particles under these different conditions, and interpret the results in a framework that combines true material properties with time-dependent effects that are governed by the permeability and the size of the individual hydrogel particles.

The focus of our work was not on the particular chemistry of the hydrogels, but rather on the fundamental behavior of these materials, which we expected to apply across many different hydrogel materials and systems. We, therefore, used poly-acrylamide, an archetypal hydrogel material [7,27,32,33,34,35], to create our hydrogel particles. In the fabrication procedure, we employed an optimized microfluidic step emulsification process to create monodisperse emulsions of aqueous droplets, which were then cross-linked internally to form uniformly sized batches of hydrogel particles. The method enabled us to reproducibly make large batches of particles, which, besides being useful in our current study of single particle mechanics, were ideally suited for creating particles for applications such as size exclusion chromatography, and drug delivery, or as a model porous material, mimicking blood flow within biological tissue.

We found that the hydrogel particles exposed to osmotic pressure had higher compression and shear moduli than the same particles in water. This effect could be attributed to the additional compression of the hydrogels by the applied osmotic pressure, which implied a higher cross-link density and shorter mesh size inside the hydrogel material.

However, for our time-dependent results, we surprisingly found a slower characteristic time scale for the PAAm particles exposed to osmotic pressure. This delay could not be accounted for, theoretically, if we considered the expected scaling for the permeability of the particle with the reduction of the particle volume imposed by osmotic pressure. However, we realized that the slow time scale could be accounted for by considering the diffusion of the osmolyte itself. The experiments on our hydrogel particles exposed to an external osmotic pressure suggested that the time-dependence of the response was, in fact, dominated by the diffusion of the osmolyte molecules within the background liquid. This suggested that, in general, when accounting for the time-dependent mechanical response of hydrogels exposed to polymer solutions, it is not sufficient to only consider the inherent poroelastic effects that generally govern the dynamics of swelling and deswelling of hydrogels. In addition, the dynamics of the macromolecules in the surrounding polymer solution need to be taken into account, as our results indicated they could dominate the time-dependence of the response.

## 2. Materials and Methods

### 2.1. Step Emulsification

To create our hydrogel particles using step emulsification, we first made stock solutions of 40 wt% Acrylamide (A8887, Sigma-Aldrich, Amsterdam, The Netherlands) and of 2 wt% N,N′-Methylenebisacrylamide (M1533, Sigma-Aldrich, Amsterdam, The Netherlands). To prepare the precursor gel solution, we added appropriate amounts of the two stock solutions and of the photoinitiator, 2-Hydroxy-67 4′-(2-hydroxyethoxy)-2-methylpropiophenone (410896, Sigma-Aldrich, Amsterdam, The Netherlands), to MilliQ water, resulting in final concentrations of 12 wt% of acrylamide monomers, 0.25 wt% of the cross-linker N,N′-methylenebisacrylamide, and 0.5 wt% of the photoinitiator, respectively.

The precursor solution was stirred until all components were fully dissolved and then degassed for 15 min to remove oxygen, which improved the crosslinking efficiency.

To achieve larger batches of particles, we employed the method of step-emulsification and we used a PDMS-based step emulsification device similar to that described by Stolovicki et al. [36]. In step-emulsification, flow of the continuous phase is not required for droplet formation, as opposed to conventional droplet making devices, which rely on viscous shear forces induced by the flow of the continuous phase fluid [36,37,38]. Instead, in step emulsification, it is the surface tension itself that drives droplet formation. The method relies on a step in the channel height, where the difference in Laplace pressure at the step leads to the formation of droplets of well-defined size. As this process is governed by surface tension instead of viscous drag forces, the size of droplets produced in these devices is very stable, remaining constant over a wide range of applied flow rates of the dispersed phase. However, in such devices, droplet accumulation at the exit can become a problem that affects the monodispersity of droplet size. To avoid this Stolovicki et al. [36] suggested using a design in which droplets are cleared at the exit aided by buoyancy, a principle that we also adopted in our step emulsification devices.

**Figure 1:**
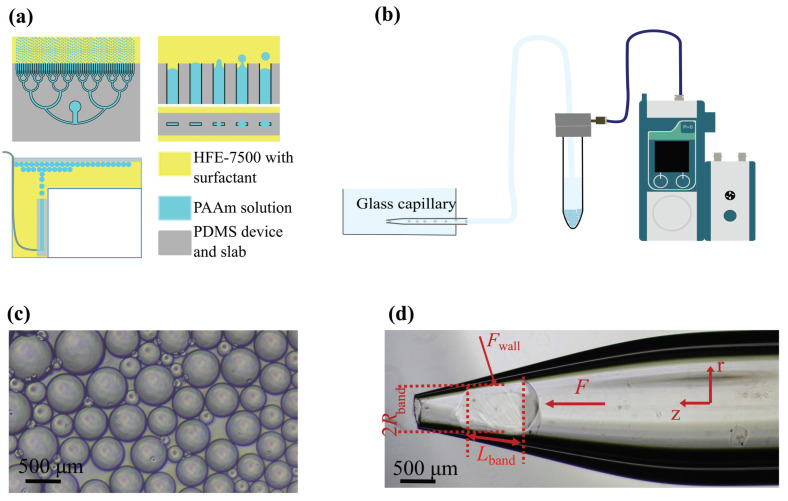
Experimental setup and schematics. (**a**) Schematic of the experimental setup for step-emulsification. The PDMS-based step-emulsification device [36] is shown in grey, the oil-phase in yellow and the polyacrylamide precursor gel solution in blue. The PDMS slab that prevented an oil–air interface can be seen on top in the bottom left image. The droplets floated to the top after exiting the nozzles of the device. (**b**) Schematic of the Capillary Micromechanics experimental setup. The Fluigent Flow EZ modular pressure controller pressurized the falcon tube with particles suspended in the aqueous dextran solution and the suspension flowed into the capillary, which was placed in a reservoir filled with a dextran solution of the same concentration. (**c**) PAAm particles made with the step-emuslification method after being suspended in water. (**d**) PAAm particle trapped in the tapering section of the capillary. Forces experienced by the particle, *F* and $F_{\mathrm{wall}}$, and the effective $L_{\mathrm{band}}$ and $R_{\mathrm{band}}$, as mentioned in Wyss et al. [27], are indicated.

We used a design consisting of 64 parallel, 700 μm wide channels with a spacing of 100 μm, as shown in Figure 1a. To fabricate the device, we used a standard photolithography process to make PDMS devices with channels of 100 μm height. To make the surface of the channels more hydrophobic, which makes the droplet formation more stable and enables high production rates, the device was surface treated by injecting a solution of 2% *w*/*v* of Trichloro (1H,1H,2H,2H-perfluoroctyl)silane (448931, Sigma-Aldrich, Amsterdam, The Netherlands) in the engineered fluid HFE-7500 (051243, Fluorochem BV, Amsterdam, The Netherlands) and the channels were left exposed to this solution for 5 min before flushing the device with water.

The whole device was submerged in a tank, as shown at the bottom left of Figure 1a with 2% *w*/*w* Fluosurf in HFE-7500 (1903-05 Emulseo, Pessac, France). A PDMS slab was placed on top of the HFE-7500 to eliminate an air–oil interface. The flow rate was set to 1.5 mL/min, using a syringe pump.The droplets floated to the surface because of their lower density and spread out evenly underneath the PDMS slab. Once the droplet formation process was finished, the entire tank was exposed to UV-light (Omnicure S-2000, 320–500 nm) for 5 min at an intensity of 8 mW/cm^2^. The cross-linked PAAm beads were subjected to a series of washing steps (see Supplementary Material) in order to transfer them from the oil phase to water.

### 2.2. Capillary Micromechanics Setup

The Capillary Micromechanics setup is shown in Figure 1b. We induced flow of a dilute suspension of PAAm particles through the system by applying a pressure difference between the inlet and the outlet of a tapered capillary [27]. The tip diameter and the length of the of the tapered capillary were controlled by tuning the processing parameters of the micropipette puller (Sutter instrument P-97). Furthermore, the tip diameter was adjusted to a suitable size, smaller than the diameter of the particles to be tested but large enough that they could be pressed through the tip opening, by using a microforge (Narishige MF2).

The pressure difference was applied by using a modular pressure-based flow controller (Fluigent Flow EZ LU-FEZ-2000), as shown in Figure 1b. Once a single particle was trapped in the tapering section of the capillary, it blocked the flow and the entire pressure difference fell across the particle. As a result, an external stress was applied to the particle and it deformed. The deformation was imaged using a Nikon Ts2R inverted microscope with a Nikon DS-Fi3 Camera, which was triggered using an Arduino Uno.

The process of image acquisition and applying a pressure difference, once the particle blocked the flow, was controlled simultaneously using a Python script. In the case of a creep test, the script initiated a step-increase in the applied pressure and captured images of particle deformation at logarithmically spaced time intervals.

**Figure 2:**
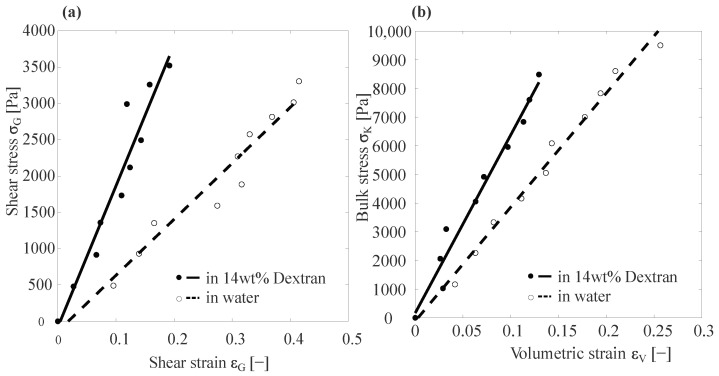
Typical stress strain curves from Capillary micromechanics. (**a**) A typical plot of shear stress as a function of shear strain, as measured by Capillary Micromechanics for a PAAm particle suspended in water (dashed lines and open circles) and suspended in 14 wt% dextran (solid line and filled circles). The slope of the fitted line is equal to the shear modulus $G_{\mathrm{water}}=7.71$ kPa and $G_{\mathrm{Dex}}=16.89$ kPa. (**b**) A typical plot of the bulk stress as a function of the volumetric strain; the slope of the fitted lines corresponds to the bulk modulus $K_{\mathrm{water}}=39.02$ kPa and $K_{\mathrm{Dex}}=62.14$ kPa.

For a basic Capillary Micromechanics measurement, the pressure was increased and the deformation of the particle imaged after waiting for the particle to reach equilibrium. The internal stresses were then computed using the equations stated in [27] and we plotted the stress as a function of strain, as shown in Figure 2.

We applied an osmotic pressure to our PAAm particles using a solution of dextran with a molecular weight of 70 kg/mol (31390, Sigma-Aldrich, Amsterdam, The Netherlands). While many types of macromolecules could have been used as an osmolyte to apply an osmotic pressure, we chose this particular grade of dextran because the molecular weight was large enough for us not to expect it to readily penetrate into the pores of the hydrogel. Moreover, the osmotic pressure of this grade of dextran had been previously characterized in detail as a function of concentration, in a range spanning from 1 wt% to 15 wt% [39]. Using the polynomial fit to the experimental data provided by Bonnet-Gonnet et al. [39], we could express the concentration-dependence of the osmotic pressure as $\Pi \left(\tilde{c}\right)=\left[286\tilde{c}+87{\tilde{c}}^{2}+5{\tilde{c}}^{3}\right]$ Pa, where $\tilde{c}$ represents the concentration in wt% normalized by 1 wt%. In our experiments, we used 14 wt% of dextran, which corresponded to an osmotic pressure of 34.7 kPa, according to the polynomial mentioned above.

For the experiments with applied osmotic pressure, we. first. flushed the entire system with dextran, shown in Figure 1b. Subsequently, we added the PAAm particles in the falcon tube and ensured that the applied pressure was at the lowest in the range of pressures we worked with in the experiment, until a particle was trapped in the tapered section of the capillary.

## 3. Results and Discussion

We recorded the response of our PAAm particles to externally applied direct forces in two cases: without osmotic pressure (PAAm particles in water) and with applied osmotic pressure (PAAm particles in a 14 wt% dextran solution).

In this section, we first show the results of the static or basic Capillary Micromechanics measurements and then we move on to the time-dependent creep test. In this section, a dashed line and an open circle indicate the response of PAAm particles in water. Furthermore, a solid line and filled circle correspond to the response of PAAm particles subjected to pre-compression using 14 wt% dextran.

### 3.1. Basic Capillary Micromechanics Measurements

We obtained the results for the static measurements using the computations/calculations mentioned in a previous article [27]. Figure 2 shows the typical stress–strain curves from measurements done using Capillary Micromechanics.

**Figure 3:**
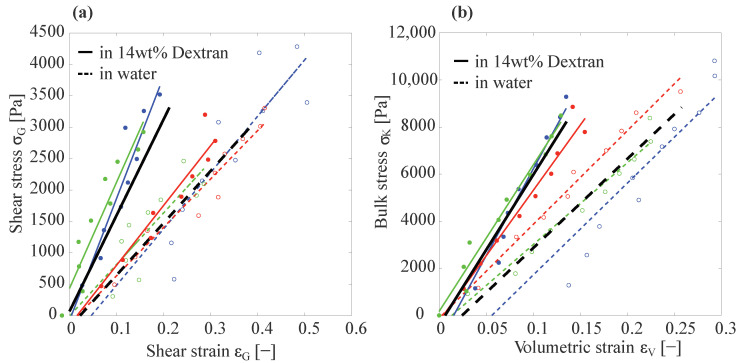
Stress–strain plots of different PAAm particles suspended in water and in 14 wt% dextran. (**a**) Plot of the shear stress as a function of shear strain for different PAAm particles suspended in water (dashed lines and open circles) and in 14 wt% dextran (solid line and filled circles) as measured by Capillary Micromechanics. The black dashed line is the average of the fitted lines for all particles measured in water and its slope represents the average shear modulus of the particles suspended in water. In analogy, the black solid line is the average of the fitted lines for particles suspended in 14 wt% dextran and its slope represents the average shear modulus of these particles. $G_{\mathrm{water}}=7.4\pm 1.9$ kPa and $G_{\mathrm{Dex}}=16\pm 5.2$ kPa. (**b**) Bulk stress as a function of volumetric strain; the slope of the dashed black line is the average bulk modulus of PAAm particles suspended in water $K_{\mathrm{water}}=36.2\pm 4.1$ kPa and the slope of the solid black line is the average bulk modulus of particles suspended in 14 wt% dextran $K_{\mathrm{Dex}}=63.4\pm 7.5$ kPa.

Figure 3 shows the shear strain versus shear stress and bulk stress as a function of volumetric strain for different PAAm particles suspended in water (dashed lines and open circles) and in 14 wt% dextran (solid lines and filled circles). In Figure 3a, the slope of the black dashed line represents the average shear elastic modulus *G* of different particles suspended in water and the slope of the black solid line represents the *G* of different particles suspended in 14 wt% dextran. $G_{\mathrm{water}}=7.35\pm 1.9$ kPa and $G_{\mathrm{Dex}}=16\pm 5.2$ kPa. Similarly, Figure 3b gives the average bulk modulus, $K_{\mathrm{water}}=36.15\pm 4.05$ kPa and $K_{\mathrm{Dex}}=63.43\pm 7.45$ kPa.

As expected, for the hydrogels subjected to an elevated osmotic pressure by the dextran solution, we found higher values for both the compressive modulus *K* and the shear modulus *G*. For *K*, we found an increase of ≈75%, while for the shear modulus *G* we observed an increase of ≈117%. Since the osmotic pressure applied by the dextran solution (≈35 kPa) was approximately equal to the average compressive modulus of the particle as measured in water (≈36.15 kPa), the PAAm particles would experience a pre-compression, due to the applied osmotic pressure of approximately a factor of two in volume. To theoretically estimate the effects of this pre-compression by a factor of two on the resultant elastic moduli, we recall that both the bulk and the shear elastic modulus are generally expected to be proportional to the inverse of the volume available to each cross-link. With the mesh size ζ being the average distance between cross-links, this implied a scaling of $K\left(\zeta \right),G\left(\zeta \right)\propto \zeta^{-3}$. As upon compression of the hydrogel material, the mesh size scaled as $\zeta \propto V^{1/3}$, this implied $K\left(\zeta \right),G\left(\zeta \right)\propto V^{-1}$ and, thus, a factor of ≈2 increase in *K* and *G* for particles compressed by the 14 wt% dextran solution, compared to those suspended in water. This was in good agreement to the observed increases in the moduli of ≈75% and ≈117% for *K* and *G*, respectively.

After performing static measurements, we conducted a series of time-dependent measurements using Capillary Micromechanics, in analogy to a so-called *creep test* in traditional rheometry, in which the time-dependent strain response to an applied shear stress was probed. We again performed these creep tests for our particles in water and in a 14 wt% dextran solution.

### 3.2. Creep Test Using Capillary Micromechanics

**Figure 4:**
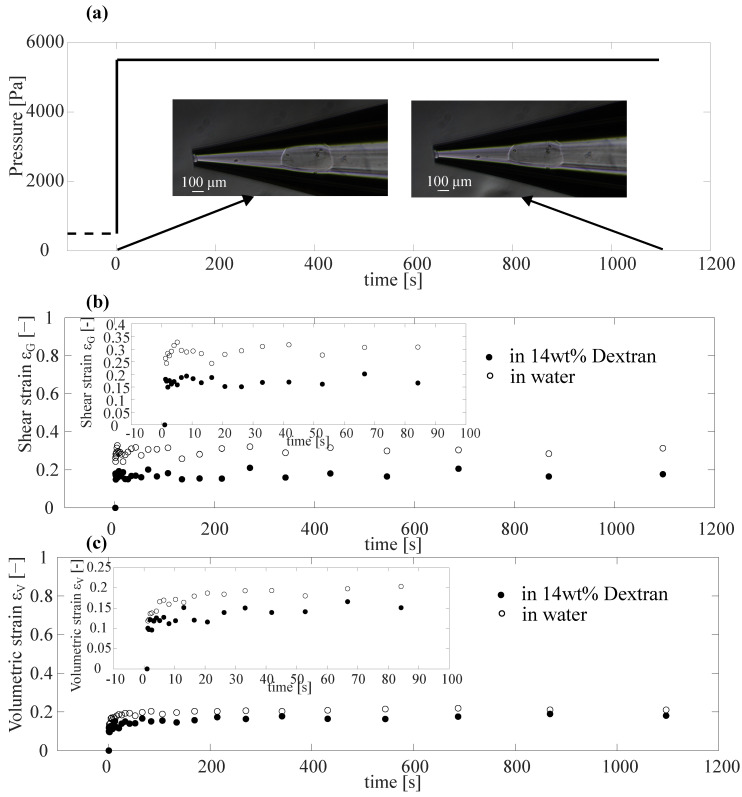
Response of PAAm particles to creep test using Capillary Micromechanics. (**a**) Applied pressure as a function of time generated using the Fluigent Flow EZ module (shown schematically in Figure 1b). The applied step-up in pressure started at an initial pressure of 500 Pa and increased to 5500 Pa. The insert microscope images show the particle in the initial and final state, respectively. (**b**) Shear strain $\epsilon_{G}$ of a PAAm particle suspended in water (open circles) and for a particle in 14 wt% dextran (filled circles) as a function of time in response to the step increase in applied pressure. The insert zooms in on the response during the first 100 s. (**c**) Volumetric strain $\epsilon_{V}$ of PAAm particle as a response to the creep test.

Once a particle was trapped inside the tapered capillary, we subjected it to a step-increase of the applied pressure, as shown in Figure 4a, and recorded the resultant changes in shear strain $\epsilon_{G}$ and volumetric strain $\epsilon_{V}$ as a function of time, as depicted in Figure 4b,c. We observed a faster response of the shear strain $\epsilon_{G}$, as compared to the volumetric strain $\epsilon_{V}$. This difference could be attributed to the fact that, for changes in volume to occur, water had to be transported in or out of the hydrogel particle, being pressed through the pores of the gel network. Any change in $\epsilon_{V}$ was, thus, hindered by the permeability of the gel network and the characteristic time scales for these volume changes were expected to depend sensitively on the size of the hydrogel particle. Conversely, a change in the shear strain $\epsilon_{G}$ did not require liquid to be pushed into or out of the hydrogel particle; as such, we would indeed expect the response of $\epsilon_{G}$ to be more rapid and less dependent on the size of the hydrogel particle. We here focused on $\epsilon_{V}$ to study the retardation time of our particles in terms of changes in their volume.

**Figure 5:**
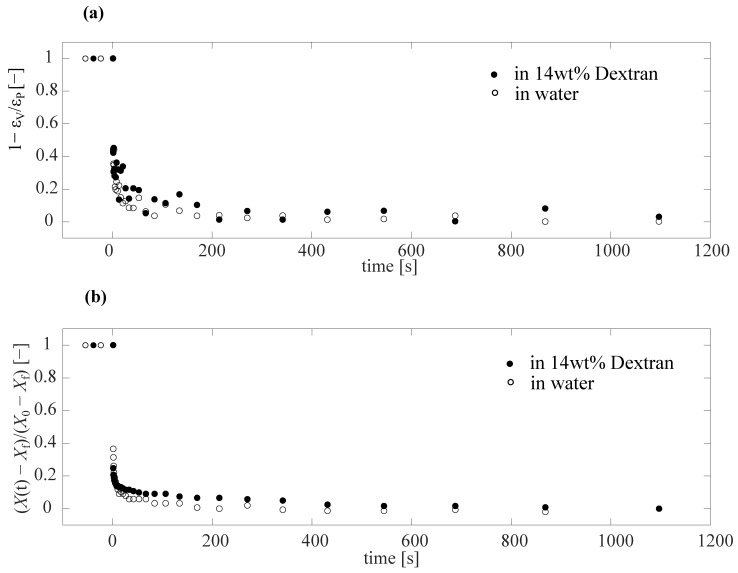
Plots to show the difference in the deformation time scale of the particle in water and 14 wt% dextran using Capillary Micromechanics. (**a**) $1-\frac{\epsilon_{V}}{\epsilon_{p}}$ as a function of time. The plateau strain $\epsilon_{p}$ represents the mean of last 5 points of the volumetric strain versus time in Figure 4c. (**b**) To reduce the noise of the data observed in (**a**), and to extract the retardation time, we plot the scaled front point position $\left(X\left(t\right)-X_{f}\right)/\left(X_{0}-X_{f}\right)$, where $X_{0}$ is the initial front point position at t=0, $X_{f}$ the final front position, and X(t) the time-dependent front position, as shown in the top schematic of Figure 6a.

To more clearly isolate the time-dependence of the response, we replotted the data of Figure 4c in a rescaled fashion in Figure 5a as $1-\epsilon_{V}/\epsilon_{p}$ as a function of time, where the plateau strain $\epsilon_{p}$ was taken as the average of the last 5 recorded points. This resulted in a decay from 1 to 0. However, it was still difficult to identify a clear difference between the data of PAAm particle suspended in water and dextran from this figure, due to noise in the data. The noise in the data arose as all the characteristic points on the image used to reconstruct the full shape and volume of the particle were selected manually, with each manual point selection being prone to statistical errors (see Figure S1 in the Supplementary Materials). To reduce these sources of error, we used an automated procedure to extract only the position of the front edge of the particle as a measure of the overall deformation of the particle. We tracked the recorded grayscale intensity along a horizontal line in the images, where the point of lowest intensity corresponded to the front point of the particle, yielding the front point position X(t). Subsequently, we normalized X(t) by subtracting the final location of the front of the particle $X_{f}$ and dividing by $X_{0}-X_{f}$, where $X_{0}$ was the initial front position of the particle. As can be clearly seen from Figure 5b, the noise in the data reduced significantly, while the overall shape was still in good agreement with the data shown in Figure 5a.

We can identify a difference in the time scale of deformation between the particles in water (open circles) and in dextran solution (filled circles). However, the particles tested did not have the same size, and the poroelastic nature of the response of hydrogels particles implies a dependence of the characteristic time scale on the size of the hydrogel, Poroelastic theory [40] suggests a scaling proportional to the square of the particle radius (see also Equation (1) below).

**Figure 6:**
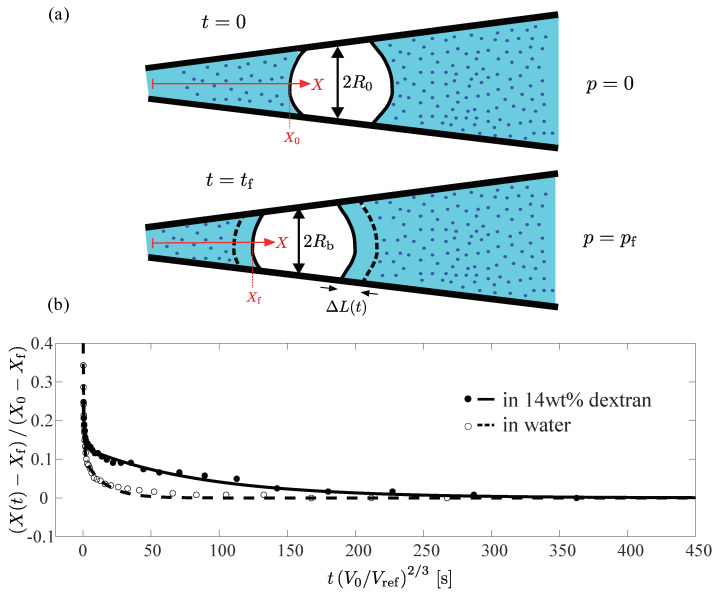
Extracting the time scale of deformation of PAAm particles in water and in a 14 wt% dextran solution. (**a**) Schematic illustration of particle deformation in dextran solution. *Top*: Hydrogel particle prior to deformation in the tapered glass capillary, surrounded by dextran osmolyte molecules (shown in dark blue) in aqueous solution. *Bottom*: The same particle in its deformed state. Due to the compression of the particle, water was pushed out, leading to the formation of an osmolyte-free zone outside the particle, as indicated by the dashed lines. (**b**) Normalized front point location as a function of the time normalized by ${\left(V_{0}/V_{\mathrm{ref}}\right)}^{2/3}$, where $V_{0}$ was the initial particle volume and $V_{\mathrm{ref}}$ was a reference volume, which we set to the average volume of the PAAm particles after fabrication. Open circles and filled circles represent the data points for PAAm particle in water and 14 wt% dextran solutions, respectively. The dashed and solid lines show double exponential fits to the data. The double exponential fit was performed, since we had an rapid initial displacement followed by a slow approach to the plateau. We identified the slower of the two time scales from the fit as the retardation time scale of the particles. The retardation time scales were ≈15 s and ≈90 s for the particle in water (dashed line and open circles) and in a 14 wt% dextran solution (solid line and filled circles), respectively.

Therefore, to compare the response of the particles on an equal footing, representative of the relative properties of the hydrogel material itself, we plotted, in Figure 6b, the normalized front point position as a function of the rescaled time scale $t{\left(V/V_{\mathrm{ref}}\right)}^{2/3}$, where $V_{\mathrm{ref}}$ was a reference volume, which we set to the average volume of the PAAm particles after fabrication. We now clearly observed a significant difference between the behaviors measured for the two different systems. While in both cases a rapid initial displacement occurred, the slower approach towards a final plateau value occurred at different characteristic time scales. For the particle in the dextran solution this approach occurred significantly slower than for the particle water.

The data clearly suggests two separate time scales for the response: an initial fast displacement of the front position, followed by a slower process. The simplest way to account for this was to fit the data to the sum of two exponential functions, where each exponential function represented a process with a single characteristic time scale. We, thus, performed double experimental fits to the data, as shown in Figure 6b as a dashed and a solid line for the particles in water and dextran, respectively. In both cases, we identified the slower of the two time scales from each double exponential fit as the retardation time scale of the particles. We found $\tau_{\mathrm{sc}.,\mathrm{water}}\approx$ 15 s and $\tau_{\mathrm{sc}.,\mathrm{dextran}}\approx$ 90 s for the rescaled characteristic time scales of the particle in water (dashed line and open circles) and in a 14 wt% dextran solution (solid line and filled circles), respectively.

### 3.3. Simple Model to Account for Creep Test Measurements

Attempting to theoretically account for this marked difference in the retardation time observed for the particles suspended in water and in the dextran solution, we considered the effects of the pre-compressed state of the particle in the dextran solution. The particle suspended in the dextran solution was exposed to a significant osmotic pressure and we, thus, expected its volume *V* to be reduced, compared to its initial volume in water, Therefore, this particle would exhibit a higher internal concentration of polymer and a higher cross-link density. The mesh size ζ, taken as the average distance between cross-links, was, thus, also shorter, which implied lower permeability of the polymer network.

To estimate the scaling of a typical deswelling time scale with the degree of pre-compression of the gel, we considered the work of Tanaka and Fillmore [40], in which an analytical solution for the kinetics of swelling of a spherical hydrogel particle is derived as a function of the initial gel radius *a*, and the viscosity η of the background liquid, as well as the elastic modulus and the permeability κ of the hydrogel material. Considering only the slowest time scale from the provided series expansion in Tanaka and Fillmore, we approximated the characteristic retardation time as

(1) $$\tau \approx \frac{a^{2}\eta }{\pi^{2}K\kappa }\;,$$

where *K* is the compressive modulus of the particle. Note that, in Figure 6, we had already accounted for the dependence on the particle radius *a*, and that the viscosity η inside the hydrogel remained unchanged for the two cases considered in Figure 6. Based on the poroelastic effects considered in the theory of Tanaka and Fillmore, we would, thus, expect the characteristic time scales extracted from the fits in Figure 6 to scale as $\tau_{\mathrm{sc}.}\propto {\left(K\kappa \right)}^{-1}$.

Expressing this scaling as a function of the mesh size of the gel network, we presumed again that $K\propto {\left(1/\zeta \right)}^{3}$, and the permeability was expected to scale with the mesh size as $\kappa \propto \zeta^{2}$, in agreement with a simple estimation of the permeability of a porous network, where the material was considered as a packing of tubes of radius ζ/2, which yielded $\kappa =\zeta^{2}/32$ (see Supplementary Material).

In combination, this yielded an expected scaling of $\tau_{\mathrm{sc}.}\propto \zeta$, which clearly suggested that, for the hydrogel pre-compressed by the dextran solution, we would actually expect a shorter characteristic time scale than for the the particles in water, i.e., $\tau_{\mathrm{sc}.,\mathrm{dextran}}<\tau_{\mathrm{sc}.,\mathrm{water}}$. This was in sharp contrast to our experiments, where we observed $\tau_{\mathrm{sc}.,\mathrm{dextran}}\approx 90$ s as compared to $\tau_{\mathrm{sc}.,\mathrm{water}}\approx 15$ s.

To account for this discrepancy, we realized that the poroelastic properties of the hydrogel material could not account for the observed change in retardation time. Instead, we focused our attention on the influence of the surrounding polymer solution on the kinetics of particle deformation. As the hydrogel was compressed, water was pressed out and replaced the dextran solution surrounding the particle in a layer of thickness ΔL(t), as illustrated schematically in Figure 6a, where we show the total layer thickness ΔL, expected to occur between the initial state where p=0 and the final, compressed state, where $p=p_{f}$. In our physical picture of the process, we hypothesized that, as the osmolyte molecules were no longer in contact with the surface of the hydrogel, the osmotic pressure was no longer acting on the particle, thus, counteracting further compression. The compression process would, thus, be gradually slowed down and the time scale for deformation would be almost completely governed by the diffusion of the dextran molecules within the liquid surrounding the particle.

The typical time scale for the dextran molecules to “catch up” with the particle surface was limited by diffusion. Therefore, to estimate the corresponding typical time scale, we considered the diffusion of dextran molecules in water, which depended on the molecular weight and the concentration of dextran. For our case, we had a molecular weight of 70 kDa and a concentration of 2 mM. Interpolating between values for the diffusion coefficient of 70 kDa dextran solutions that were measured at various concentrations by Albro et al. [41], we found a value of $D\approx 10\;\mu m^{2}/s$. The typical time scale for diffusion of the dextran molecules over the layer thickness ΔL was then

(2) $$\tau_{D}\approx \frac{{\Delta L}^{2}}{2D}\;.$$

With the total layer thickness as $\Delta L\approx \left(V_{0}\epsilon_{V}\right)/\left(2\pi {R_{0}}^{2}\right)$, with $\epsilon_{V}\approx$ 18%, $V_{0}\;\approx \;14.8\times 10^{6}\;{\mu m}^{2}/s$, and $R_{0}\approx 110\;\mu m$, we found ΔL≈35μm. Using a diffusion coefficient of $D=10\;\mu m^{2}/$s, we finally extracted a diffusion time scale of $\tau_{D}\approx 61$ s.

Given the simplicity of our model description, this estimated characteristic time scale for diffusion was in remarkably good agreement with the retardation time measured in our experiments on the particles in the dextran solutions, $\tau_{\mathrm{sc}.,\mathrm{dextran}}\approx 90$ s, and it accounted for the observed slow-down of the particle compression dynamics compared to that observed for particles in pure water.

We, thus, hypothesized that, for the dextran system, the typical time scale of deformation was governed by the diffusion of dextran molecules in the bulk liquid surrounding the particle, rather than by the transport of liquid through the pores of the hydrogel network.

While this physical picture already accounted for our experimental data, a possible further explanation for the observed slow-down of compression dynamics for particles exposed to dextran solutions could be that dextran molecules might have penetrated into the pores of the hydrogel in the time period before the creep tests were performed. This would increase the effective viscosity inside the hydrogel and, therefore, also lead to a slow-down of the particle deformation, compared to the case without dextran added to the background liquid. However, as shown in our previous work [7,9], if the dextran macromolecules could fully penetrate the hydrogel, this would lead to a slow reswelling of the particles to their initial volume, with minimal effect on their elastic moduli *K* and *G*. However, when we exposed our particles to dextran, we observed a noticeable increase in their elastic moduli. This suggested that dextran did not significantly penetrate the hydrogel network in the system being studied here. In Sleeboom et al. [7], where we observed penetration of osmolyte into the gel network, we ahd used the same dextran molecules as osmolyte and PAAm particles with lower polymer and cross-linker concentrations; the mesh size of these gels was, thus, significantly larger. For the particles used here, we, therefore, did not expect a significant penetration of dextran into the gel network. Nevertheless, even without considering this potential additional effect, we were able to account for the behavior observed in our experiments using a simple model picture, based solely on the influence of the diffusion of the osmolyte molecules within the surrounding liquid.

## 4. Conclusions

We measured the static and time-dependent responses of single hydrogel particles using Capillary Micromechanics, and compared both the static and time-dependent responses of systems with, and without, an additionally applied osmotic pressure. For the static measurements, as expected, we found an increase in the shear and compressive moduli of the particles subjected to pre-compression by osmotic pressure. However, for the time-dependent response, we unexpectedly found a slower time scale for the PAAm particles exposed to osmotic pressure. This delay could not be theoretically accounted for by considering the scaling of the permeability of the particle with its volume reduction due to osmotic pressure, as poroelastic theory would imply the opposite effect. However, a simple model applied to our experiments on hydrogel particles suggested that this slower response was, in fact, governed by the diffusion of the osmolyte molecules in the background liquid that surrounded the particles. This showed that, in general, when accounting for the time-dependent mechanical response of hydrogels exposed to polymer solutions it is not sufficient to only consider the inherent poroelastic effects that generally govern the dynamics of swelling and deswelling of hydrogels. The dynamics of the macromolecules in the surrounding polymer solution need to be, additionally, taken into account, as our results indicated they could dominate the time-dependence of the mechanical response.

## Supplementary Materials

A supporting information manuscript can be downloaded at: https://www.mdpi.com/article/10.3390/gels9030194/s1, including section 1: Image analysis for Capillary Micromechanics; section 2: Full dataset for shear and volumetric strain as a function of time; section 3: Full dataset for normalized point location as a function of time; section 4: Double exponential fit for PAAm particles in water and 14 wt% Dextran; section 5: Washing protocol for transferring particles from oil to water phase; Figure S1: Point selection for image analysis of Capillary Micromechanics; Figure S2: Full dataset for shear and volumetric strain as a function of time in response to a creep test; Figure S3: Full dataset for normalized front point location; Figure S4: Full dataset of samples used for extracting retardation time scales using double exponential fits; Table S1: Parameters for double exponential fits to data in Figure S4.
