# Supplementary material for: Single Hydrogel Particle Mechanics and Dynamics Studied by Combining Capillary Micromechanics with Osmotic Compression
Source: Gels. 2023 Mar 3;9(3):194. doi: 10.3390/gels9030194 (PMC10048562; doi:10.3390/gels9030194)
Supplement: Supplementary file 1 [file gels-09-00194-s001.zip › SuppMat.pdf]

Supplementary Materials: Single Hydrogel Particle Mechanics and Dynamics Studied by Combining Capillary Micromechanics with Osmotic Compression

Kalpit J. Bakal, Andreas M. A. O. Pollet, Jaap M. J. den Toonder and Hans M. Wyss

1. Image analysis for Capillary Micromechanics

To extract the changes in shape and volume of the particles from the microscopy images, we use ImageJ to manually select characteristic points as shown in figure S1. Point 1 is a reference point and the coordinates of all other points are processed relative to the position of point 1 (we use this to correct for any changes in position of the glass capillary within the field of view that might occur during measurements; if the sample does not move at all between frames, then we use a fixed value for position 1 in all frames to reduce errors). We select these points for all images in the stress-strain tests and the creep test; the associated errors in manually identifying these points contribute to the noise in the data from the creep test in figure S2. Similarly, these errors contribute to the data shown in figure 3 in the main text. See also the error analysis provided in our original article on Capillary Micromechanics [1].

(a)

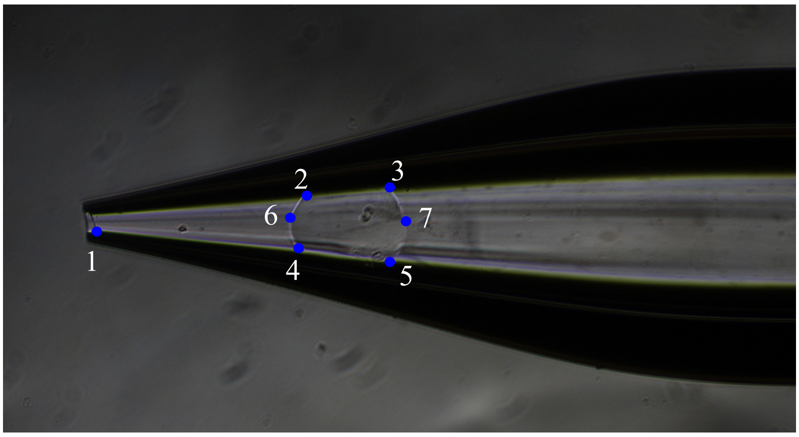

(b)

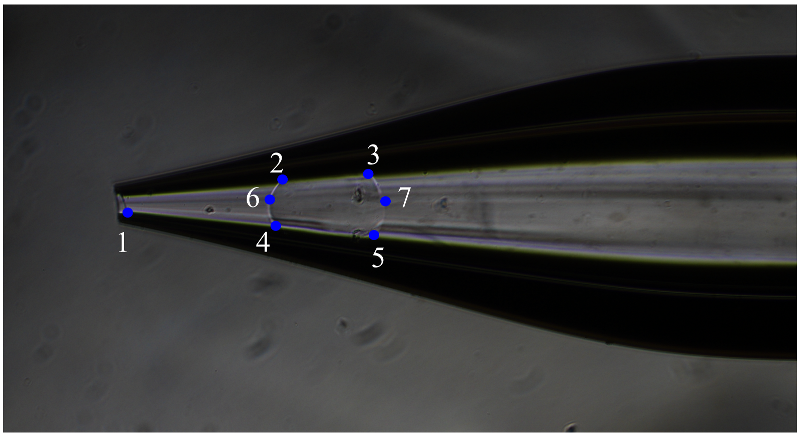

Figure S1. *Point selection for image analysis of Capillary Micromechanics* **(a)** Image of the particle immediatelay after it is trapped in the tapered section of the glass capillary at the beginning of the measurement. Point 1 is the reference point relative to which all other points are measured. The order of the point selection is kept consistent for all the images. **(b)** Image of the particle in the final position of the creep test, with the same manually selected characteristic points. We clearly observe a change in shape as well as volume of the particle compared to the initial image.

2. Full dataset for shear and volumetric strain as a function of time

We have performed a range of independent experiments on different particles, to test the repeatability of our measurements. Figure S2 shows the full dataset for the shear strain and volumetric strain as a function of time in a creep test as shown in the main manuscript in figure 4(a). The noisiness in the data arises mainly due to the errors associated with the manual point selection, perforemd as illustrated in figure S1.

We find that the general behavior in terms of the characteristic time scale of deformation is consistent between the different experiments for both the experiments performed in the dextran solution (filled circles) and for particles in water (open circles), while the magnitude of the final strains varies more markedly between the different experiments. When performing the same analysis as in the main manuscript, tracking the front point position of each particle to extract the time-dependent deformation of the particle with less associated noise, we find a much closer agreeement, as shown in section 3 below.

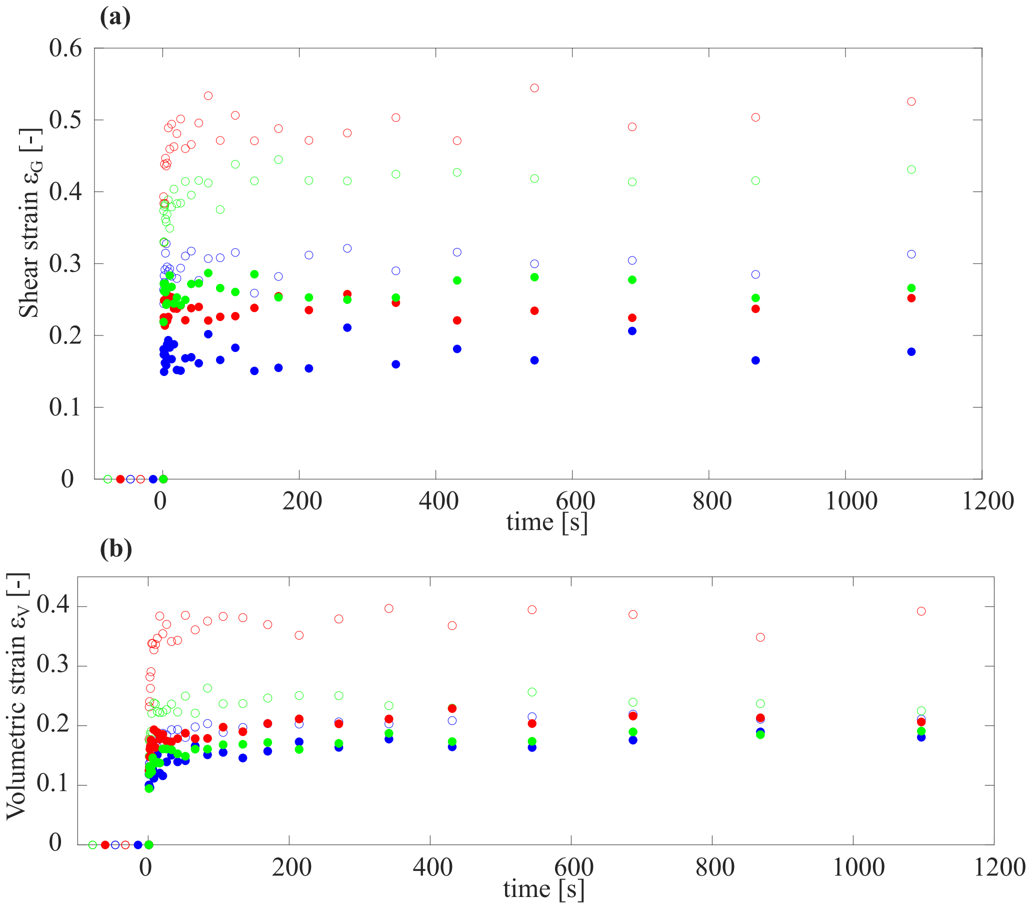

Figure S2. *Full dataset for shear and volumetric strain as a function of time in response to a creep test* **(a)** Shear strain ϵ_G response as a function of time in response to the creep test. Open circles and filled circles indicate Polyacrylamide PAAm particles in water and in 14 wt% Dextran, respectively. **(b)** Volumetric strain ϵ_V response as a function of time in response to the creep test. Open circles and filled circles indicate Polyacrylamide PAAm particles in water and in 14 wt% Dextran, respectively.

3. Full dataset for normalized point location as a function of time

For the same experiments shown in section 2, we also performed the analysis explained in the main text, where we track the front point location of the particle in order to extract the time-dependence of the response while reducing the noise in the data. We track the front point location of the particle, denoted by point 5 in figure S1(a) and (b) along a horizontal line, by locating the minimum value of the intensity along this line. The front point location is normalized by subtracting the final front point location of the particle X_f from the current location $X(t)$ and dividing by $X_0 - X_f$, where X_0 is the initial particle location.
We find a consistent time dependence for the particles in water (open circles) and for the particles in the dextran solution (filled circles).

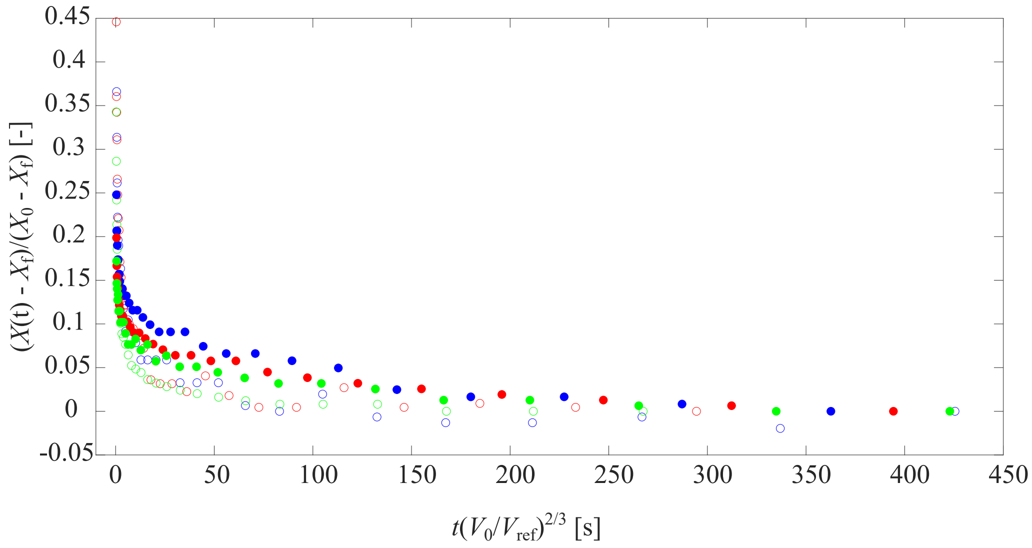

Figure S3. *Full dataset for normalized front point location.* The normalized front point location as explained in figure 5(b) and figure 6(c) as a function of the normalized time $t(V_0/V_{\mathrm{ref}})$, as explained in figure 6(c). Filled circles represent PAAm particles in 14 wt% Dextran and open cirlces represent the PAAm particles in water.

4. Double exponential fit for PAAm particles in water and 14wt % Dextran

For the same plot shown in section 3, we fit the data using a double exponential fit. As explained in the main text, we extract the retardation time from the slower of the two associated time scales. We use the expression in equation 1, where t_1 represents $t(V_o/V_{\mathrm{ref}})^{2/3}$ and y represents $(X(\mathrm{t}) - X_{\mathrm{f}})/(X_0 - X_{\mathrm{f}})$. The resultant parameters of the double exponential fit can be found in table S1. In the table, W1-W3 stands for the particles suspended in water, and D1-D3 stands for the particles suspended in 14wt % Dextran. The parameter τ_2 represents the slowest timescale for each fit; we clearly observe that the retardation time τ_2 is higher for the samples in dextran than for those in water.

$$y = ae^{-t_1/\tau_1} + be^{-t_1/\tau_2} \tag{1}$$

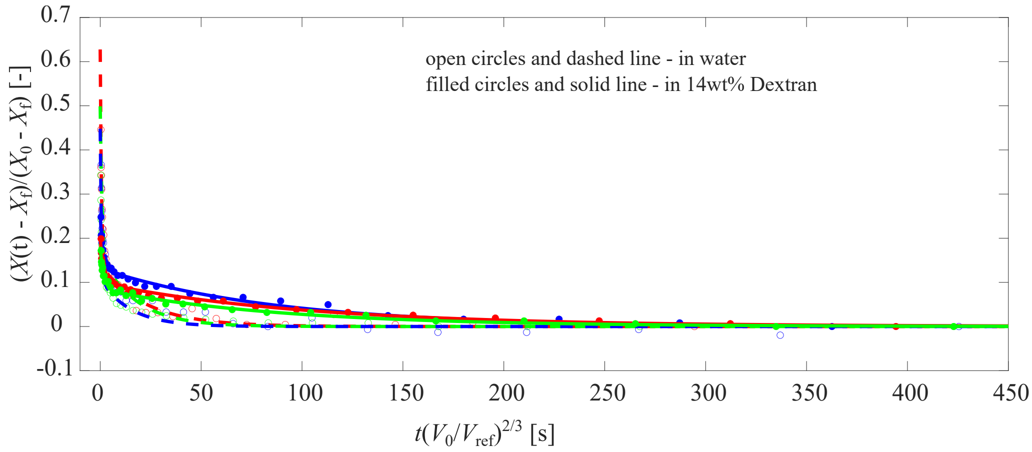

Figure S4. *Full dataset of samples used for extracting retardation time scales using double exponential fits.* The double exponential fit as explained in figure 6(b) in the main manuscript. Open circles and dashed lines represent the PAAm particles in water and filled circles and solid lines represent the particles in 14wt % Dextran. We measured 3 PAAm particles in water and 3 PAAm particles in 14wt % Dextran and find a fair agreement in behavior for both datasets.

Table S1. Parameters for double exponential fit for equation 1.

PAAm particle	a	b	τ_1[s]	τ_2[s]
W1	0.4879	0.1397	0.613	22.49
W2	0.3359	0.1629	0.877	17.39
W3	0.3408	0.1072	0.619	15.01
D1	0.1234	0.1312	1.2	86.91
D2	0.1076	0.0984	1.28	103.5
D3	0.0963	0.0842	1.737	89.79

5. Washing protocol for transferring particles from oil to water phase

Washing protocols followed for PAAm particles made in HFE-7500 oil suspended in a falcon tube. The protocol was provided by Dr. Elisa Mele.

- Centrifuge the falcon tube at 3000g for 30s. Remove the bottom HFE-7500 oil phase.
- Add 500 μL of neat HFE-7500 followed. Vortex and centrifuge the falcon tube and remove the bottom oil phase.
- Add 500 μL of HFE-7500 with 20%(by volume) fluorooctane. Vortex and centrifuge and remove the bottom oil phase.
- Add 1 mL 1% span-80 in Hexanes to the falcon tube. Vortex and centrifuge and remove the upper Hexane phase.
- Add 1 mL 1% neat Hexanes to the falcon tube. Vortex and centrifuge and remove the upper Hexane phase.
- Add 1 mL 0.1% triton X-100 in water. Vortex and centrifuge and remove upper water phase including any oil water emulsion floating on top of the liquid.
- Add 1 mL 0.1% triton X-100 in water. Pipette up and down, vortex.
- The particles are ready to be dispersed in water

Reference

1. Wyss, H.M.; Franke, T.; Mele, E.; Weitz, D.A. Capillary micromechanics: Measuring the elasticity of microscopic soft objects. *Soft Matter* **2010**, *6*, 4550–4555.
