# Supplementary figures and images for: Single Hydrogel Particle Mechanics and Dynamics Studied by Combining Capillary Micromechanics with Osmotic Compression
Source: Gels. 2023 Mar 3;9(3):194. doi: 10.3390/gels9030194 (PMC10048562; doi:10.3390/gels9030194)

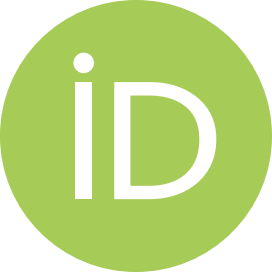

Supplement: Supplementary file 1 [file gels-09-00194-s001.zip › Definitions/logo-orcid.pdf]

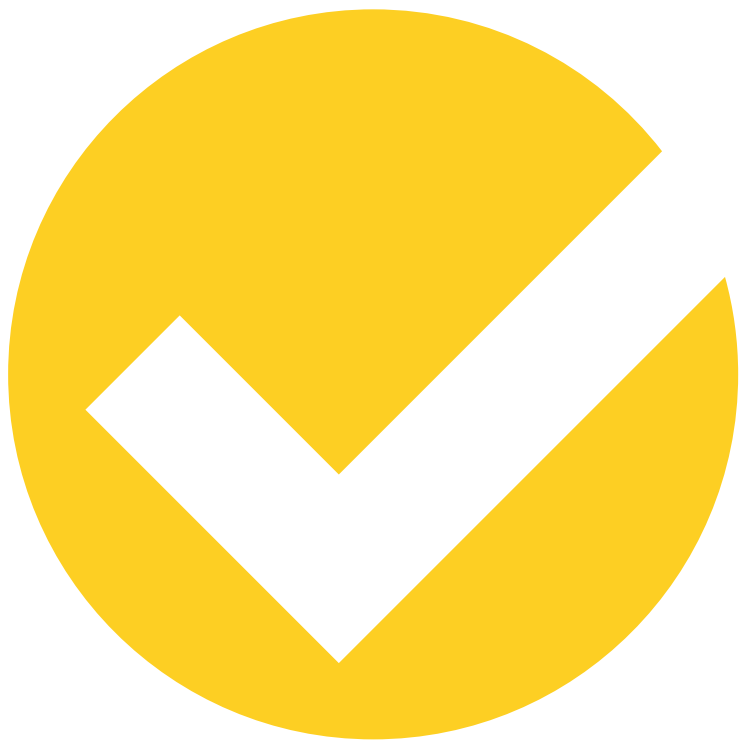

check for  
updates

Supplement: Supplementary file 1 [file gels-09-00194-s001.zip › Definitions/logo-updates.pdf]

**(a)**

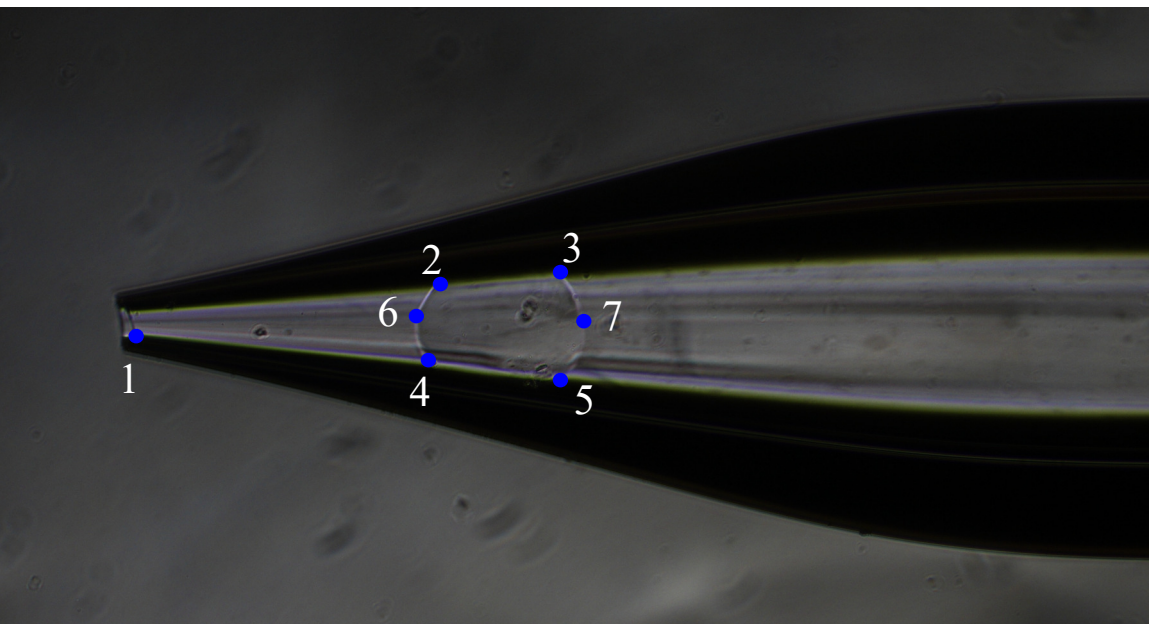

**(b)**

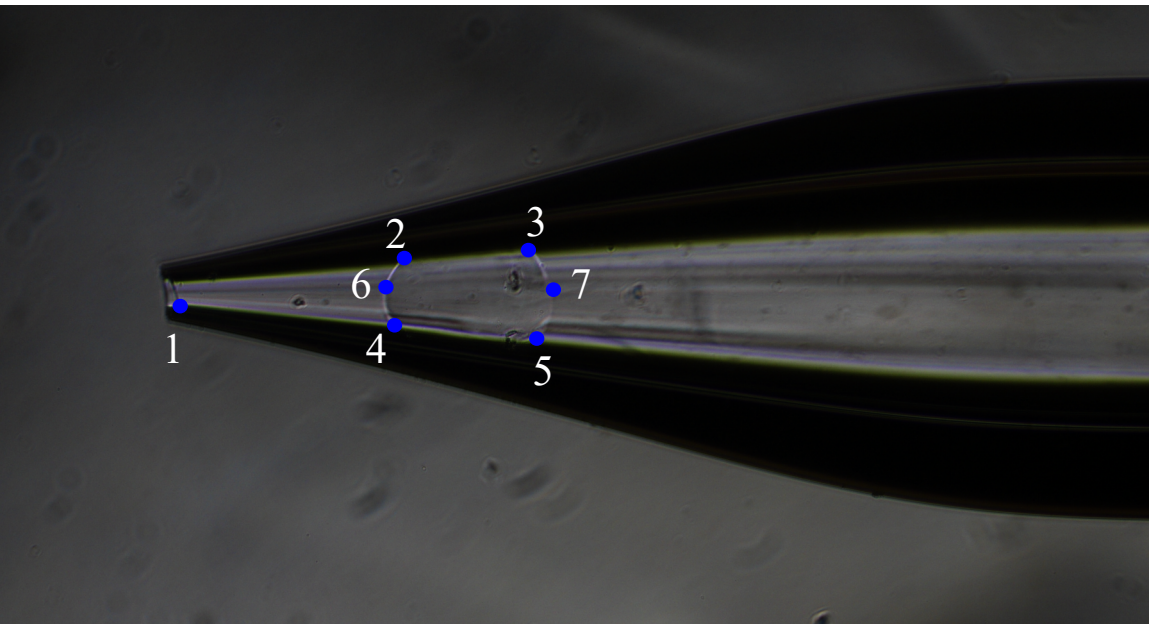

Supplement: Supplementary file 1 [file gels-09-00194-s001.zip › Figure_S1-eps-converted-to.pdf]

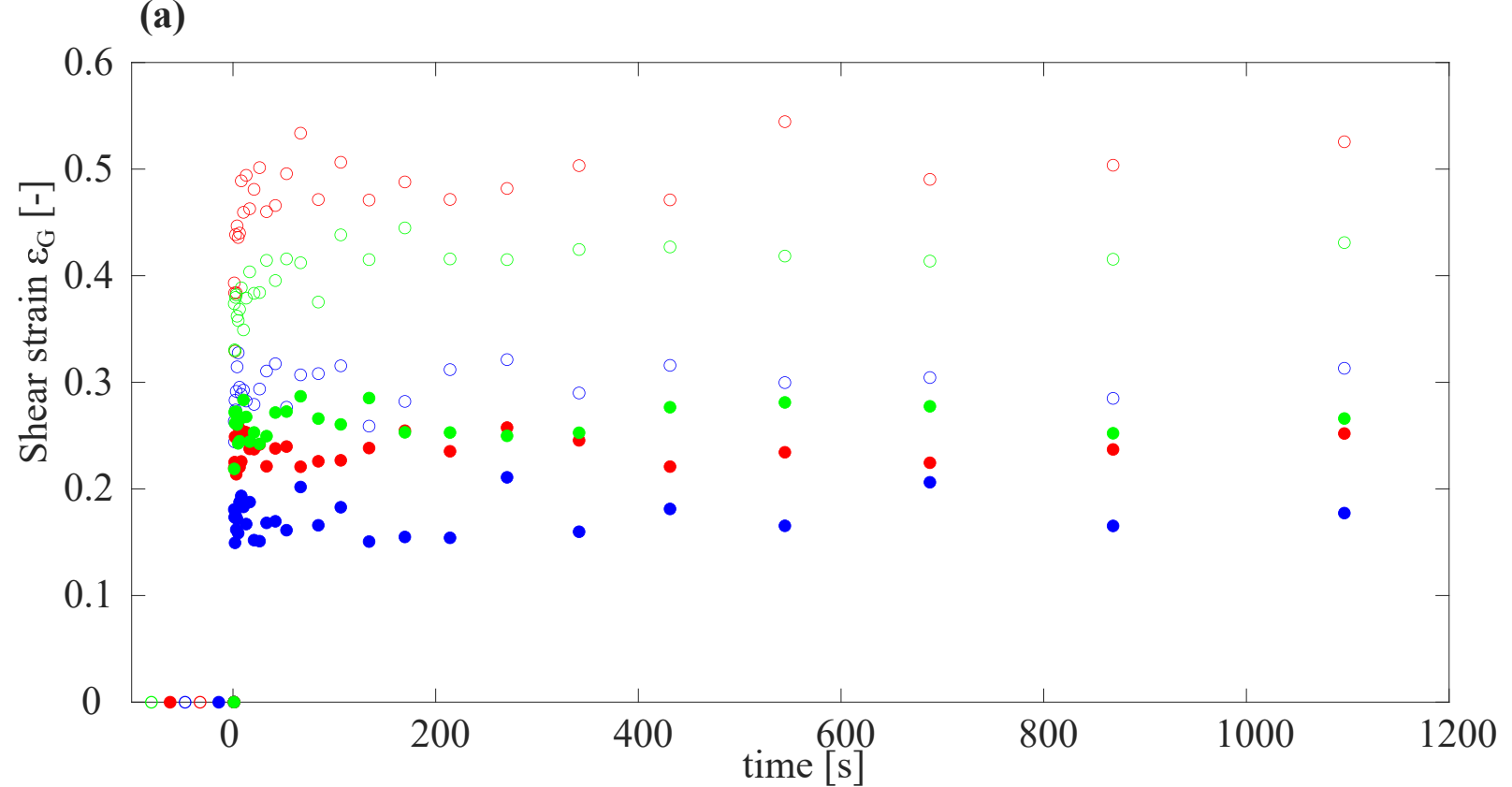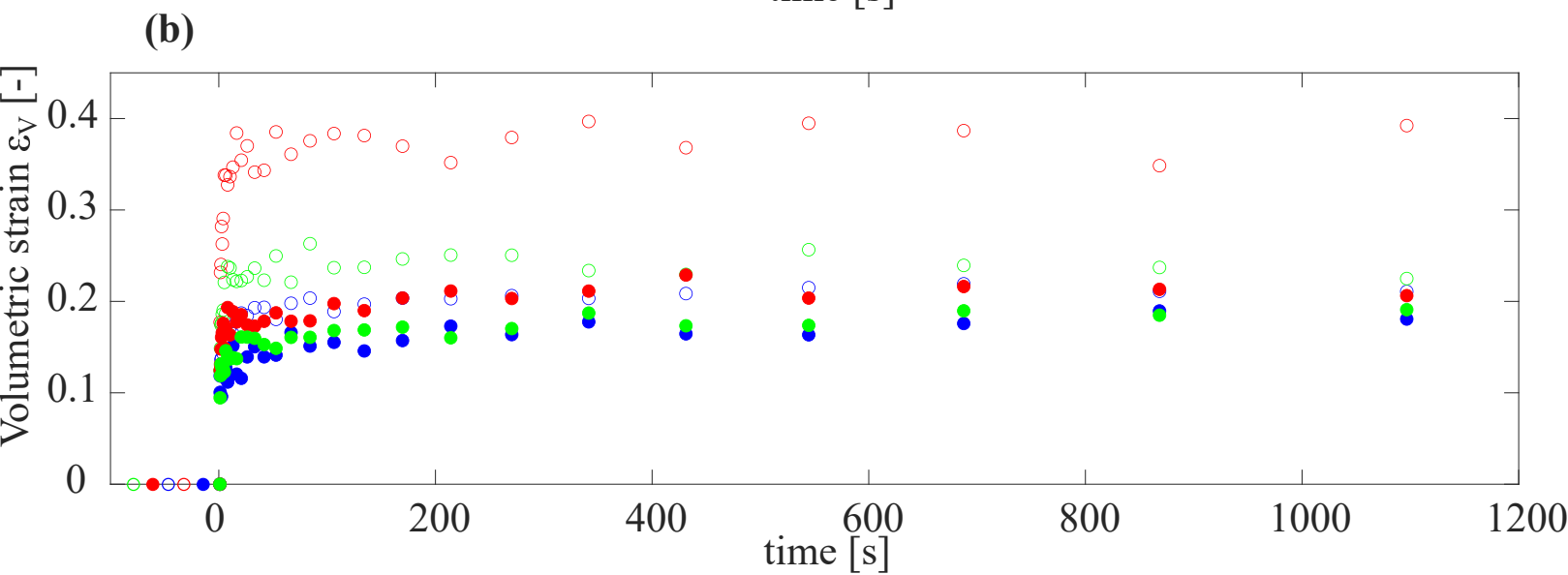

Supplement: Supplementary file 1 [file gels-09-00194-s001.zip › Figure_S2-eps-converted-to.pdf]

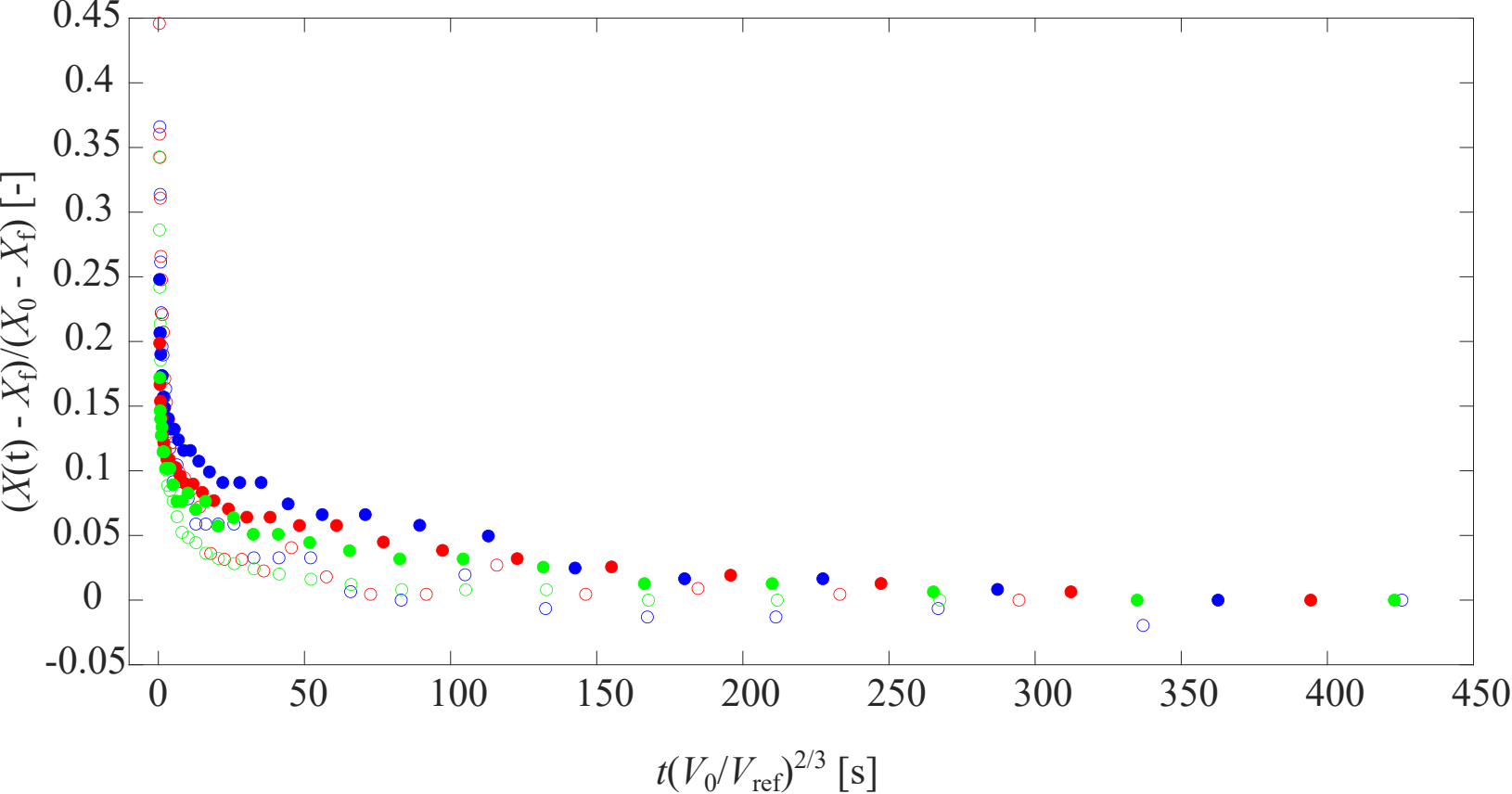

Supplement: Supplementary file 1 [file gels-09-00194-s001.zip › Figure_S3-eps-converted-to.pdf]

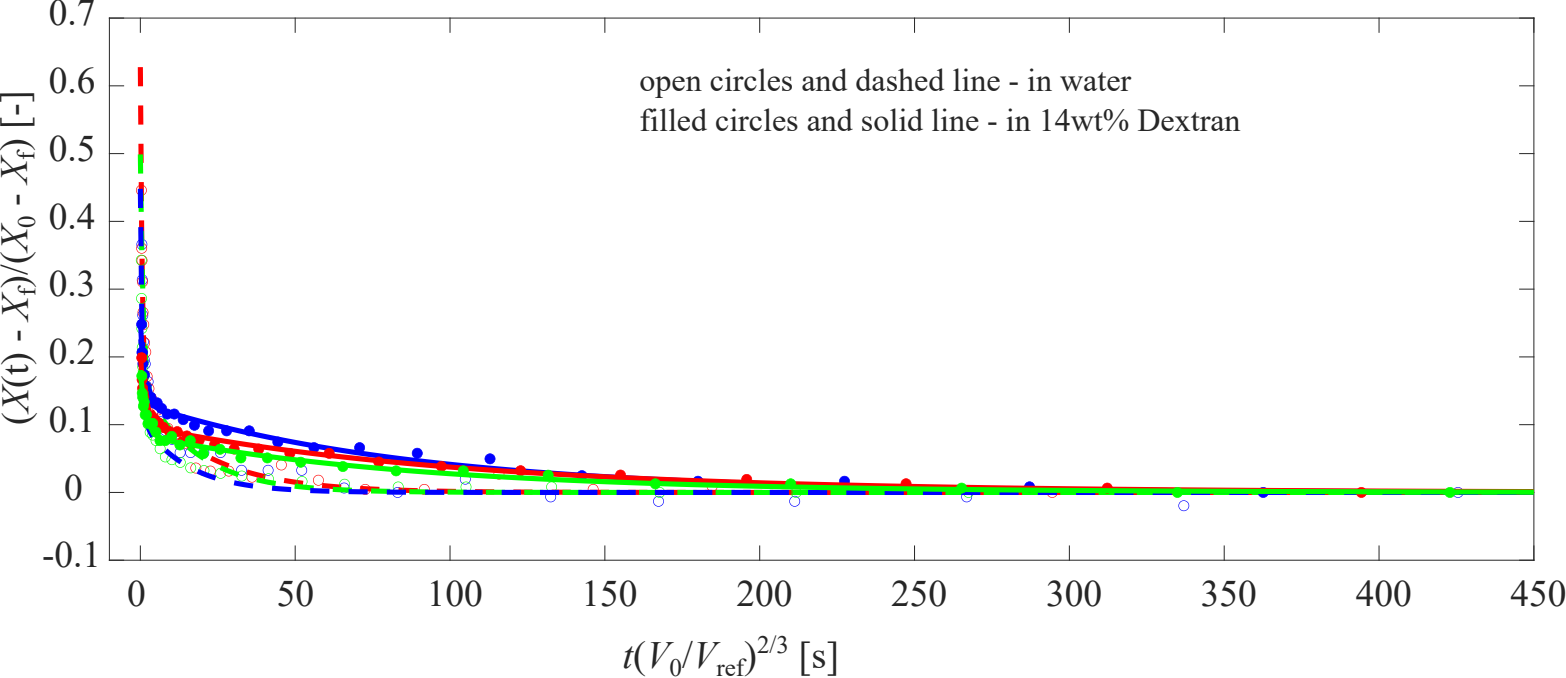

Supplement: Supplementary file 1 [file gels-09-00194-s001.zip › Figure_S4-eps-converted-to.pdf]
